# Pathological complete response to neoadjuvant tislelizumab plus chemotherapy in stage IIIB small cell lung cancer: A case report and literature review

**DOI:** 10.3389/fimmu.2023.1111325

**Published:** 2023-02-22

**Authors:** Nan Zhou, Yuhong Chen, Qian Huang, Lili Jiang, Hu Liao, Hongfeng Gou, You Lu, Guowei Che, Yan Zhang

**Affiliations:** ^1^ Lung Cancer Center, West China Hospital, Sichuan University, Chengdu, Sichuan, China; ^2^ Gastric Cancer Center, Division of Medical Oncology, Cancer Center, Laboratory of Gastric Cancer, State Key Laboratory of Biotherapy, West China Hospital, Sichuan University, Chengdu, Sichuan, China; ^3^ Department of Abdominal Oncology, West China Hospital, West China Medical School, Sichuan University, Chengdu, Sichuan, China; ^4^ Division of Medical Oncology, Cancer Center, West China Hospital, Sichuan University, Chengdu, Sichuan, China; ^5^ Department of Pathology, West China Hospital, West China Medical School, Sichuan University, Chengdu, China; ^6^ Department of Thoracic surgery, West China Hospital, West China Medical School, Sichuan University, Chengdu, China; ^7^ Department of Thoracic Oncology, Cancer Center, West China Hospital, West China School of Medicine, Sichuan University, Chengdu, Sichuan, China

**Keywords:** small cell lung cancer, neoadjuvant therapy, pathological complete response, immunotherapy, tislelizumab, programmed cell death-1 (PD-1), lymphocyte infiltration, case report

## Abstract

Immunotherapy plus chemotherapy has been approved for the first-line treatment of extensive-stage small cell lung cancer (ES-SCLC, stage IV). Recently, the 2023 version of the National Comprehensive Cancer Network Guidelines recommended immunotherapy plus chemotherapy as the neoadjuvant regimen in patients with resectable non-small cell lung cancer (NSCLC). However, it is still unclear whether the combination regimen of immunotherapy plus chemotherapy is also beneficial for SCLC in the neoadjuvant context. Here, we report the case of a patient with stage IIIB SCLC who showed long-term survival and good tolerance to the neoadjuvant chemoimmunotherapy consisting of tislelizumab (an anti–PD-1 monoclonal antibody) plus etoposide-carboplatin. The patient achieved pathological complete response after receiving two cycles of neoadjuvant tislelizumab and chemotherapy followed by surgery. Two courses of post-operative tislelizumab and etoposide-carboplatin treatment were performed. The patient has survived for more than 23 months with no recurrence or metastases after neoadjuvant therapy. Multiplexed immunofluorescence and immunohistochemistry staining showed that the post-treatment specimens had remarkable immune cells infiltration, including CD3+ T cells, CD4+ T cells, and CD8+ T cells, which contrasted with very low levels of these cells in the pre-treatment samples. This study is, to the best of our knowledge, the first attempt to present the neoadjuvant chemoimmunotherapy of tislelizumab in combination with etoposide-carboplatin in SCLC. Our study suggested that neoadjuvant tislelizumab plus chemotherapy may facilitate radical resection and benefit patients with locally advanced (stage IIB-IIIC) SCLC.

## Introduction

It is generally acknowledged that surgery is limited to very early stages (I–IIA) in small cell lung cancer (SCLC). The standard therapies for locally advanced (stage IIB-IIIC) SCLC have shown limited benefits. Therefore, improvements in treatment regimens are still needed. Neoadjuvant treatment showed promise with respect to the R0 resection (no residual tumor) rate and pathological complete response (pCR, 0% viable tumor in resected lung and lymph nodes) rate, which are related to clinical benefits (1–4). However, the pCR rate was as low as 5% (2/40) for patients with stage IIIA SCLC who were treated with neoadjuvant chemotherapy alone (5).

Tislelizumab is a monoclonal antibody with the high affinity for PD-1. A phase II study revealed potent antitumor effects of tislelizumab plus platinum-etoposide in extensive-stage small cell lung cancer (ES-SCLC, stage IV), with a median PFS of 6.9 months and 1-year overall survival rate of 76% (6).

Previous studies have demonstrated that chemotherapy enhances antitumor activities through direct or indirect immune-system activation (7). It has been established that a combination regimen of immunotherapy and chemotherapy has superior benefits both in neoadjuvant and first-line setting for non-small cell lung cancer (NSCLC) (8–10). Tislelizumab plus chemotherapy as the first-line regimen has been approved by the National Medical Products Administration (NMPA) of China for the treatment of advanced NSCLC. In addition, the IMpower133 and CASPIAN study revealed a significant effect on OS with the addition of immunotherapy to standard chemotherapy, leading to the approval of combination regimens by the Food and Drug Administration (FDA) for first-line treatment of ES-SCLC (11, 12).

In the neoadjuvant context, immunotherapy aims to enhance systemic immunity, eliminating micrometastatic tumor deposits (13). The FDA approved nivolumab (an anti–PD-1 antibody) plus chemotherapy as the neoadjuvant regimen for resectable NSCLC based on the CheckMate-816 trial. This study showed that neoadjuvant nivolumab plus chemotherapy resulted in significantly longer event-free survival and a higher pCR rate than chemotherapy alone (2).

However, it remains unknown whether neoadjuvant immunotherapy plus chemotherapy could also be beneficial for limited-stage SCLC (LS-SCLC, stages I–III). Here, we report a patient with stage IIIB (cT3N2M0) SCLC who achieved pCR after receiving neoadjuvant tislelizumab plus etoposide-carboplatin.

## Case report

**Figure 1:**
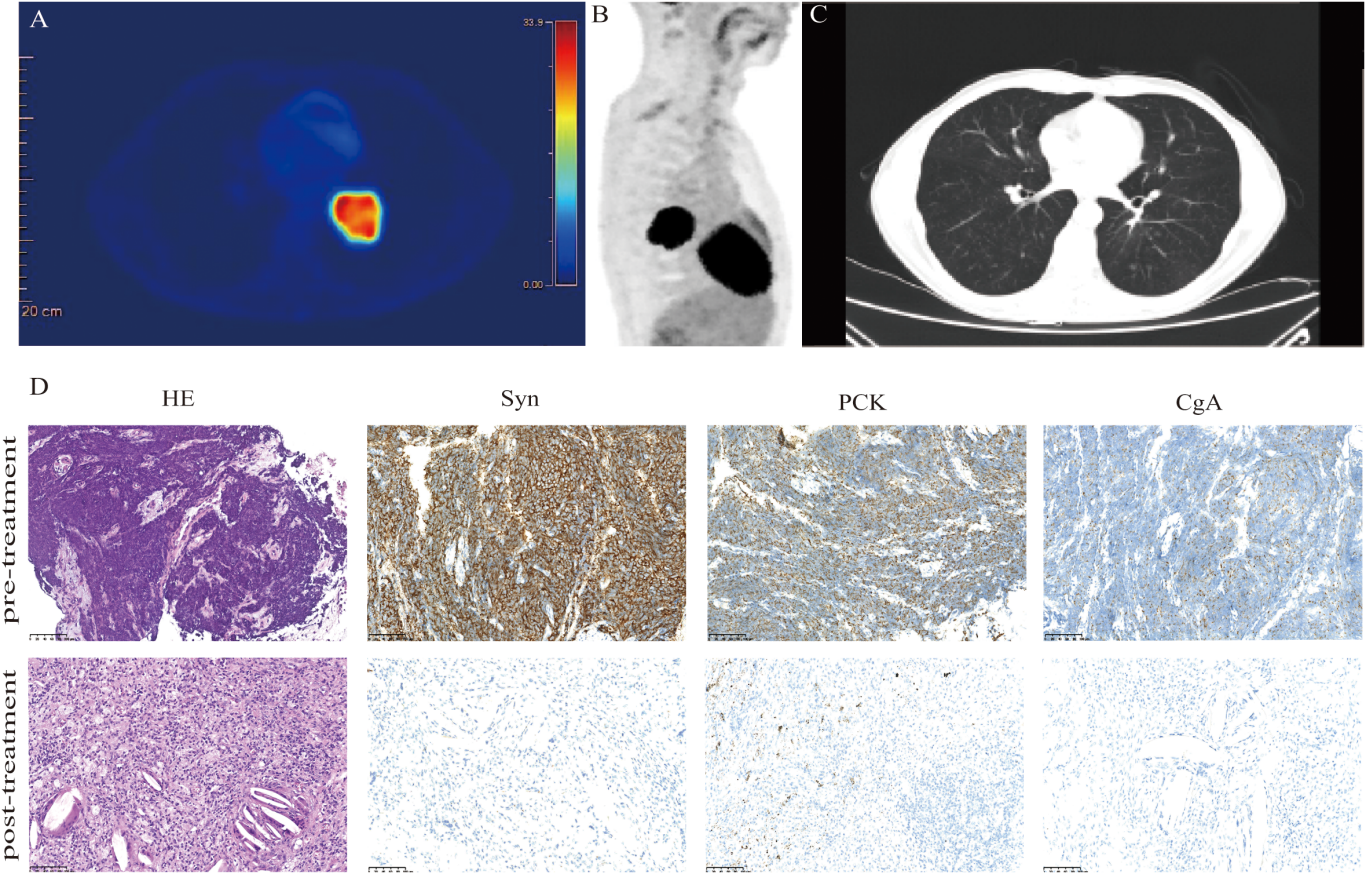
Clinical and pathological response. Images **(A, B)** show FDG uptake in the lung parenchyma. **(C)** CT images acquired after two circles of neoadjuvant tislelizumab plus chemotherapy display a decrease in the size of the tumor lesion. **(D)** Hematoxylin-eosin (HE) and immunohistochemistry staining in the tumor bed. The staining of the lung mass (lower panels) showed no viable tumor cells; negative for synaptophysin (Syn), pan-cytokeratin (PCK), and chromogranin A (CgA). Scale bars: D = 100 µm.

In January 2021, a 46-year-old Chinese man was admitted to our hospital for cough. The patient was in good health and had no history of smoking, family history of hereditary disease, or tumor. However, the chest computed tomography (CT) and positron emission tomography (PET)-CT scan presented a mass of 5.4 cm × 4.5 cm, centrally located in the left lower lobe, with hilar and mediastinal lymph nodes metastasis (**Figures 1A, B**). Blood tests showed a significant elevation of pro-gastrin-releasing peptide (Pro-GRP). The contrast-enhanced CT of the abdomen and magnetic resonance imaging (MRI) of the brain were normal. Then, the patient accepted the fiberoptic bronchoscopy and biopsy. Eventually, the diagnosis of LS-SCLC, cT3N2M0, and stage IIIB (Eastern Cooperative Oncology Group performance-status score of 0) was given. Immunohistochemical analyses suggested “PCK(+), CK7 (+), TTF-1 (+), CD56 (+), CgA (+), Syn (+), CD34(-), desmin(-)、P63(-), Ki-67 (80%+). ”

**Figure 2:**
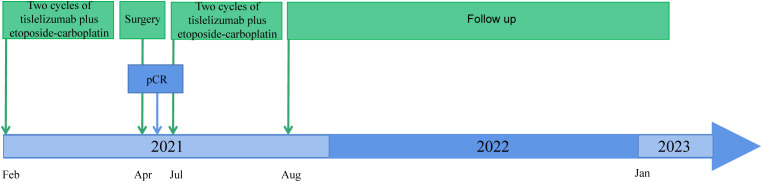
Timeline from January 2021 to January 2023.

The treatment of the patient was discussed by the multi-disciplinary team (MDT). The tumor was considered potentially resectable; thus, neoadjuvant therapy followed by surgery might be an ideal option. Our patient chose the neoadjuvant regimen of etoposide (100 mg/m^2^, days 1–3), carboplatin (AUC 5, day 1), and tislelizumab (200 mg, day 1) every 3 weeks. After two cycles of neoadjuvant treatment, a repeated CT scan manifested the mass obviously shrank to a nodule with a diameter of 0.8 cm (**Figure 1C**). Patients underwent left lower pulmonary lobectomy and lymphadenectomy 38 days after neoadjuvant tislelizumab plus chemotherapy. Notably, the efficacy was evaluated as pCR. Tumor cell was found neither in hematoxylin-eosin (HE) staining nor in immunohistochemistry (IHC) staining of pan-cytokeratin (PCK), chromogranin A (CgA), and synaptophysin (Syn) (**Figure 1D**). After surgery, the patient received two cycles of tislelizumab in combination with etoposide-carboplatin. Regular imaging assessments were done every 2 months during the follow-up period. More than 23 months after neoadjuvant tislelizumab plus chemotherapy, no recurrence or metastases were detected (**Figure 2**). The treatment-related adverse event was hypothyroidism, which was diagnosed in April 2022. No adverse events led to discontinuation.

## Discussion

We report a case of a patient with stage IIIB SCLC who obtained pCR and long-term benefits after neoadjuvant tislelizumab plus etoposide-carboplatin followed by surgery. To our knowledge, this is the first report of neoadjuvant therapy comprising tislelizumab plus chemotherapy in SCLC.

It is well accepted that significantly more immune effector T cells were in LS-SCLC than in ES-SCLC (14), suggesting that patients with earlier stage SCLC may gain more benefits from neoadjuvant chemoimmunotherapy.

**Figure 3:**
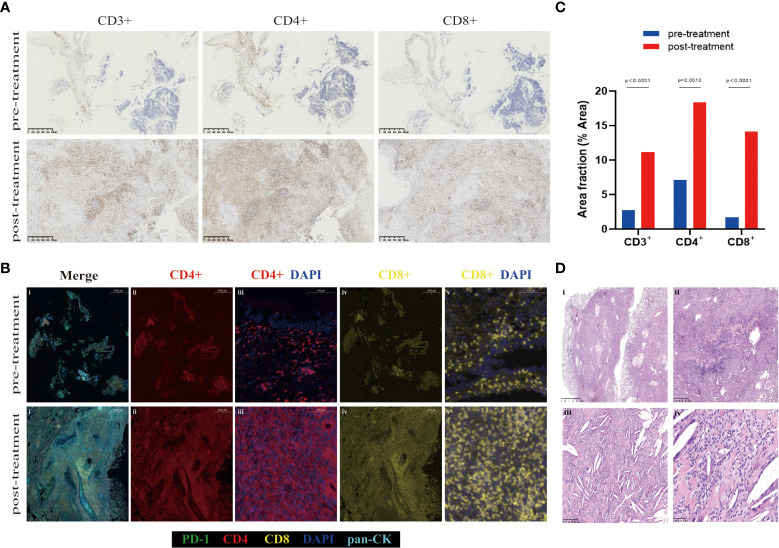
Representative images demonstrating changes in the tumor bed. **(A)** CD3+, CD4+, and CD8+ immunohistochemistry staining of the pre- and post-treatment specimen. **(B)** Multiplex immunofluorescent images of tumor tissue sections, which labels five immune biomarkers within the tumor bed: PD-1 (green), CD4 (red), CD8 (yellow), DAPI (blue), pan-CK (light blue). Overall, the numbers of various immune cell phenotypes were higher in post-treatment specimens. **(C)** Area fraction of indicated immune cell populations in pre- and post-treatment. A significant increase in CD3+, CD4+, and CD8+ in the tumor bed is observed after neoadjuvant tislelizumab plus chemotherapy (multiple unpaired *t*-tests *p*<0.000, *p* = 0.0013, and *p*<0.000, respectively). **(D)** HE staining of post-treatment specimen shows inflammatory cells, a proliferation of fibrosis, cholesterol clefts, and granulomatous reaction after neoadjuvant tislelizumab plus chemotherapy. Scale bars: A = 500 µm. B: i, ii, and iv =1000 µm; iii and v = 100 µm. D: i = 2.5 mm; ii = 500 µm; iii = 250 µm; iv = 50 µm.

The addition of immunotherapy to chemotherapy had the potential advantage of inducing systemic tumor-specific T-cell response (15). Various studies have identified that CD4+ and CD8+ T-cell subpopulations experience a proliferative burst after immunotherapy, which was associated with improved survival (16–18). To explore the tumor immune microenvironment, we performed IHC staining of CD3+, CD4+, and CD8+ T cells (**Figure 3A**) and multiplexed immunofluorescence staining of immune markers (CD4+, CD8+, DAPI, PD-1, and PanCK) (**Figure 3B**) on pre- and post-treatment specimens. Notably, the post-treatment samples exhibited remarkable immune cell infiltration. The positive area fractions of CD3+, CD4+, and CD8+ T cell in post-treatment specimens were 4.02, 2.60, and 8.38 times higher than those in pre-treatment specimens, respectively (**Figure 3C**).

Pathologic features of pCR after neoadjuvant tislelizumab plus etoposide-carboplatin therapy were observed. It was detected that inflammatory cells, a proliferation of fibrosis, cholesterol clefts, and granulomatous reaction were in the areas of the previous tumor bed (**Figure 3D**). The association of pCR with survival benefits warrants further evaluation involving patients with SCLC.

Atezolizumab or durvalumab (anti–PD-L1 antibody) in combination with chemotherapy is considered the standard of care in the first-line setting in SCLC. However, in January 2021, the patient finally selected tislelizumab (anti–PD-1 antibody), mainly due to financial distress. Of note, serplulimab, an anti–PD-1 antibody, combined with chemotherapy have recently come under intense focus after a phase 3 study showed improvement in overall survival compared with chemotherapy alone (15.4 months *vs.* 10.9 months, respectively; hazard ratio, 0.63) in patients with ES-SCLC (19). Based on this study, serplulimab was approved by the NMPA of China for the first-line treatment of ES-SCLC and was also granted orphan drug designations by the FDA and European Commission for the treatment of SCLC. Our study suggests that tislelizumab may have similar antitumor effects in SCLC and deserves further investigation.

## Conclusion

Our case shows that neoadjuvant tislelizumab plus chemotherapy might facilitate radical resection and benefit patients with locally advanced (stage IIB-IIIC) SCLC without impeding the feasibility of surgery or increasing the incidence of adverse events.

## Data availability statement

The original contributions presented in the study are included in the article/supplementary material. Further inquiries can be directed to the corresponding author.

## Ethics statement

The studies involving human participant was reviewed and approved by The Clinical Research Ethics Committee of West China Hospital Sichuan University. The patient/participant provided his written informed consent to participate in this study. Written informed consent was obtained from the individual for the publication of any potentially identifiable images or data included in this article.

## Author contributions

NZ: Conceptualization, Writing- Original draft preparation. YC: Methodology, Software. QH: Investigation. GC, HG: Visualization, Investigation. LJ: histopathological findings. YL, HL: Software, Validation. YZ: Writing- Reviewing and Editing, Supervision. All listed authors participated meaningfully in the study and that they have seen and approved the final manuscript.
